# Is religiosity/spirituality in patients with Crohn's disease important to their quality of life?

**DOI:** 10.1016/j.clinsp.2024.100389

**Published:** 2024-05-24

**Authors:** José Luiz Amuratti Gonçalves, José Jukemura, Carolina Bortolozzo Graciolli Facanali, Carlos Frederico Sparapan Marques, Rodrigo Ambar Filho, Carlos Walter Sobrado, Sergio Carlos Nahas

**Affiliations:** Departamento de Gastroenterologia, Hospital das Clínicas, Faculdade de Medicina da Universidade de São Paulo (HCFMUSP), São Paulo, SP, Brazil

**Keywords:** Quality of life, Spirituality, Crohn's disease, Chronic disease, Inflammatory bowel diseases, Coping

## Abstract

The authors aim to study Religiosity/Spirituality (R/S) and Quality of Life (QoL) in patients with Crohn's disease and their correlation with the disease phenotypes.

**Methods:**

Prospective cross-sectional cohort study with 151 consecutive patients enrolled from March 2021 to October 2021 at the Colorectal IBD Outpatient of Hospital das Clínicas da Faculdade de Medicina da Universidade de São Paulo (HCFMUSP). Sociodemographic, Religiosity/Spirituality (Duke University Religion Index – Durel) questionnaires and QoL (Inflammatory Bowel Disease Questionnaire – Short IBDQ-S) were applied. When necessary, qualitative variables were evaluated using the chi-square or Fisher's exact test. The Mann-Whitney and Kruskall-Wallis tests were used to analyze quantitative variables and compare more than two groups, both non-parametric statistical techniques.

**Results:**

The most frequent location was Ileocolonic followed by Ileal and colonic (41.1 %, 27.2 %, and 25.2 %); only 6.6 % of subjects had a perianal presentation. Inflammatory, stenosing, and penetrating behaviors showed 36.4 %, 19.1 %, and 44.4 % respectively. The majority of the population is Catholic, Evangelical, or Spiritualist (92.4 %). QoL score showed no significant difference in the phenotypes. The scores for DUREL domains were 61.4 % for organizational religiosity, 75 % for non-organizational religiosity, 98.6 %, 93.6 % and 89.3 % for intrinsic spirituality, with high results in all disease phenotypes.

**Conclusions:**

The studied population presented homogeneous sociodemographic results and high religious and spiritual activity. R/S in a positive context were not associated with better QoL or phenotype. R/S is present in the patients’ lives and could be seen as an important tool for adherence to treatment and the professional relationship between doctor and patient. The homogeneity of the sample difficult for an appropriate evaluation, which leads us to suggest new studies with more heterogeneous groups.

## Highlight

• Religiosity and spirituality in Inflammatory Bowel Disease should be further explored, being an important part of quality of life in the treatment goals of these patients.
• Our article demonstrates that the need to evaluate the relationship between quality of life and religiosity and spirituality must be carried out in heterogeneous samples in order to be statistically significant.
• The quality of life in the different phenotypes of Crohn's disease is not statistically significant

## Introduction

Crohn's Disease (CD) is one of the most important presentations of Inflammatory Bowel Disease. Clinically characterized for being a chronic disease with periods of exacerbation represented by episodes of diarrhea several times a day with elimination of blood and mucus, abdominal cramps, weight loss, and logically very compromised quality of life.^1^, ^2^, ^3^

Its etiology has been increasingly elucidated, and it is estimated that genetically predisposed individuals and dysregulated immune response (innate and acquired) associated with microbiota antigens and environmental factors such as smoking and inadequate diet are the basis for triggers in the first and subsequent flare-ups. These triggers may have their origins in several areas: emotional, losses, infections, surgeries, etc.^1^, ^2^, ^3^, ^4^

Regarding histological and morphological presentation, the CD presents discontinuous lesions in the gastrointestinal mucosa, which can affect from the mouth to the anus, in a transmural process covering all layers of the organ, causing erosions, ulcerations, stenosis, formation of fistulas that communicate with other intestinal segments, other organs such as the bladder and vagina and the abdominal wall.^4^

In addition to digestive manifestations, individuals with CD may have extra-intestinal manifestations and the most common are rheumatological, dermatological and ophthalmological.^5^

The increase in incidence and prevalence is a fact, especially in newly industrialized areas in South America, Asia and Africa. This growth follows the higher rate of urbanization, occidental diet, smoking, sedentary lifestyle, and low rates of breastfeeding. The same scenario is found in Brazil and Europe.^6^, ^7^, ^8^

When the patient is diagnosed with a chronic, debilitating, and incurable disease faces the uncertainties and questions that must be answered by the assistant team. It is observed that the patient makes use of tools in the behavioral-cognitive area called coping, which varies according to their experience and understanding. Studies conclude that religiosity and spirituality find a place of action both in acceptance and adherence to treatment and clearly in quality of life.

The authors conceptualize religiosity as the practice of a religion, which is defined as a system of beliefs and practices carried out by a community, supported by rituals that recognize, venerate, and communicate with the Sacred and the Divine.

Spirituality is an individual's quest to understand existential issues (e.g., the end and meaning of life) and its relationship to the divine and/or transcendent and does not necessarily lead the patient to develop the practice of spirituality.^9^, ^10^, ^11^, ^12^, ^13^

Because of the rising number of studies relating R/S feelings to chronic disease coping and having doctors and healthcare professionals aware of their role in supporting patients to achieve greater adherence to treatment.^14^^,^^15^ the authors' interests are to understand its importance to the different clinical presentations of Crohn´s disease.

The objective of this article is to study the quality of life of patients with Crohn's disease and understand the influence of Religiosity/Spirituality as an associated factor with the better quality of life of these patients and its relationship with the clinical presentation of the disease.

## Methods

The study was carried out at the IBD outpatient clinic of the Coloproctology discipline of Hospital das Clínicas da FMUSP (HCFMUSP), from March to October 2021, with 151 consecutive patients.

The following inclusion criteria were: age 18 and 80 years with Crohn's disease diagnosed and classified in remission or mild to moderate activity by the Harvey-Bradshaw Index, with or without previous intestinal surgery, whose data were extracted from the medical records at the time of the study.

Exclusion criteria were: individuals with diagnostic doubts, unspecific and undetermined colitis, HIV carriers, patients with no cognitive capacity to fill out questionnaires, patients with severe comorbidities such as neurological, psychiatric, cardiovascular, pulmonary, renal failure, pregnant women, lactating women, patients with hypothyroidism, neoplasms and decompensated diabetes.

All patients signed the Free and Informed Consent Term (ICF).

Patients were classified with Harvey-Bradshaw Index and the Montreal Classification and S-IBDQ (Short ‒ Inflammatory Bowel Disease Questionnaire) and p-DUREL (Duke University Questionnaire – Religiosity) questionnaires were applied, both validated for the Portuguese language.

Qualitative variables were evaluated using the Chi-Square test or Fisher's exact test when necessary. The Mann-Whitney test was used to analyze quantitative variables and to compare more than 2 groups, the Kruskall-Wallis test was used, both non-parametric statistical techniques.^16^^,^^17^

## Results

This study investigated 151 patients, 84 (56.6 %) were female and 67 (44.4 %). The age distribution is presented with a mean age of 45.2 years (±12.2-years). The socio-demographic evaluation questionnaire showed that the distribution of the individual's level of education is homogeneous from elementary to higher education. The most common income in the sample was 72.6 % up to 3 minimum wages, with married marital status being the most common, followed by single. Yet 92.4 % of the patients declared they belonged to a religion (Evangelical, Catholic, Spiritism and Jehovah's Witness).

The authors included in the questionnaire an assessment of emotional triggers and other areas (surgeries, diseases, other events) that could influence the onset of CD or its exacerbation and the result reported positively for these events was 81.7 % of respondents. Regarding leisure activities, a decrease of 20 % was detected (Table 1, Table 2, Table 3, Table 4).

**Table 1:** DC Location (segment) and Behaviour.

			IC9 5 %	
Location (Segment)	N	%	Inferior	Superior
*Ileal L1*	41	27.2%	20.5 %	34.6 %
*Colonic L2*	38	25.2 %	18.8 %	32.5 %
*Ileocolônic L3*	62	41.1 %	33.4 %	49.0 %
*Upper Gastro intestnal L4*	0	0	0	0
*Perianal (exclusive)*	10	6.6 %	3.5 %	11.4 %
Inflammatory Behaviour B1	55	36.4 %	29.1 %	44.3 %
Stenosing Behaviour B2	44	29.1 %	22.3 %	36.7 %
Penetrating Behaviour B3	67	44.4 %	36.6 %	52.3 %
PeriAnal Behaviour B3 p	62	41.1 %	33.4 %	49.0 %

For better visualization of the following Tables, the authors transcribe the content of the questions of the DUREL Religiosity/Spirituality Index:

1. How often do you go to a church, temple or other religious gathering? (Assessment of organizational religiosity).
2. How often do you dedicate your time to individual religious activities, such as prayers, prayers, meditations, reading the bible or other religious texts? (Assessment of non-organizational religiosity).
3. In my life, I feel the presence of God (or the Holy Spirit). (Assessment of intrinsic religiosity, spirituality).
4. My religious beliefs are really behind my whole way of living. (Assessment of intrinsic religiosity, spirituality).
5. I try very hard to live my religion in all aspects of life. (Assessment of intrinsic religiosity, spirituality).

The following Table 1, Table 2, Table 3, Table 4 bring us the distributions among the DC locations and behaviours (Table 1), answers to the quality of life IBDQ – short version (Table 2), the distribution answers in DUREL – religiosity and spirituality of Duke university scale (Table 3) and the Table 4 shows the distribution of the answers on DUREL table according to the DC location:

**Table 2:** Distribution of the answers and medium+Standard deviation to each category of the IBDQ (Short Version) Quality of life.

			IC95 %		Grade
	N	%	Inferior	Superior	Medium±SD
Question 1 SIBDQ					
*Always*	19	13.8 %	8.8 %	20.2 %	4 ± 2
*Almost Always*	25	18.1 %	12.4 %	25.2 %	
*Many times*	23	16.7 %	11.2 %	23.5 %	
*Few times*	25	18.1 %	12.4 %	25.2 %	
*Very Few Times*	15	10.9 %	6.5 %	16.9 %	
*Seldom*	15	10.9 %	6.5 %	16.9 %	
*Never*	16	11.6 %	7.1 %	17.7 %	
Question 2 SIBDQ					
*Always*	2	1.4 %	0.3 %	4.6 %	5 ± 2
*Almost Always*	17	12.3 %	7.6 %	18.6 %	
*Many times*	28	20.3 %	14.2 %	27.6 %	
*Few times*	25	18.1 %	12.4 %	25.2 %	
*Very Few Times*	11	8.0 %	4.3 %	13.4 %	
*Seldom*	14	10.1 %	5.9 %	16.0 %	
*Never*	41	29.7 %	22.6 %	37.7 %	
Question 3 SIBDQ					
*Always*	15	10.9 %	6.5 %	16.9 %	4 ± 2
*Almost Always*	19	13.8 %	8.8 %	20.2 %	
*Many times*	32	23.2 %	16.8 %	30.7 %	
*Few times*	19	13.8 %	8.8 %	20.2 %	
*Very Few Times*	7	5.1 %	2.3 %	9.7 %	
*Seldom*	22	15.9 %	10.6 %	22.7 %	
*==Never*	24	17.4 %	11.8 %	24.4 %	
Question 4 SIBDQ					
*Always*	22	15.9 %	10.6 %	22.7 %	4 ± 2
*Almost Always*	19	13.8 %	8.8 %	20.2 %	
*Many times*	21	15.2 %	10.0 %	21.9 %	
*Few times*	24	17.4 %	11.8 %	24.4 %	
*Very Few Times*	11	8.0 %	4.3 %	13.4 %	
*Seldom*	15	10.9 %	6.5 %	16.9 %	
*Never*	26	18.8 %	13.0 %	26.0 %	
Question 5 SIBDQ					
*Always*	13	9.4 %	5.4 %	15.1 %	4 ± 2
*Almost Always*	14	10.1 %	5.9 %	16.0 %	
*Many times*	42	30.4 %	23.2 %	38.5 %	
*Few times*	15	10.9 %	6.5 %	16.9 %	
*Very Few Times*	9	6.5 %	3.3 %	11.6 %	
*Seldom*	16	11,6 %	7,1 %	17,7 %	
*Never*	29	21.0 %	14.9 %	28.4 %	
Question 6 SIBDQ					
*Always*	36	26.3 %	19.5 %	34.1 %	3 ± 2
*Almost Always*	9	6.6 %	3.3 %	11.6 %	
*Many times*	33	24.1 %	17.5 %	31.7 %	
*Few times*	23	16.8 %	11.3 %	23.7 %	
*Very Few Times*	15	10.9 %	6.5 %	17.0 %	
*Seldom*	9	6.6 %	3.3 %	11.6 %	
*Never*	12	8.8 %	4.9 %	14.4 %	
Question 7 SIBDQ					
*Always*	26	19.0 %	13.1 %	26.2 %	4 ± 5
*Almost Always*	18	13.1 %	8.3 %	19.5 %	
*Many times*	25	18,2 %	12,5 %	25,3 %	
*Few times*	20	14.6 %	9.4 %	21.2 %	
*Very Few Times*	9	6.6 %	3.3 %	11.6 %	
*Seldom*	12	8.8 %	4.9 %	14.4 %	
*Never*	26	19.0 %	13.1 %	26.2 %	
Question 8 SIBDQ					
*Always*	10	7.3 %	3.8 %	12.6 %	4 ± 2
*Almost Always*	9	6.6 %	3.3 %	11.6 %	
*Many times*	19	13.9 %	8.9 %	20.4 %	
*Few times*	33	24.1 %	17.5 %	31.7 %	
*Very Few Times*	24	17.5 %	11.9 %	24.5 %	
*Seldom*	19	13.9 %	8.9 %	20.4 %	
*Never*	23	16.8 %	11.3 %	23.7 %	
Question 9 SIBDQ					
*Always*	18	13.1 %	8.3 %	19.5 %	4 ± 2
*Almost Always*	22	16.1 %	10.6 %	22.9 %	
*Many times*	23	16.8 %	11.3 %	23.7 %	
*Few times*	29	21,2 %	15,0 %	28,6 %	
*Very Few Times*	5	3.6 %	1.4 %	7.8 %	
*Seldom*	13	9.5 %	5.4 %	15.2 %	
*Never*	27	19.7 %	13.7 %	27.0 %	
Question 10 SIBDQ					
*Always*	27	19.9 %	13.8 %	27.1 %	4 ± 2
*Almost Always*	19	14.0 %	8.9 %	20.5 %	
*Many times*	30	22.1 %	15.7 %	29.6 %	
*Few times*	19	14.0 %	8.9 %	20.5 %	
*Very Few Times*	10	7.4 %	3.8 %	12.7 %	
*Seldom*	14	10.3 %	6.0 %	16.2 %	
*Never*	17	12.5 %	7.7 %	18.8 %	

*There may be “missing data”.

**Table 3:** Distribution of the answers on each question of DUREL.

			IC95 %		Grade*
	N	%	Inferior	Superior	Medium±SD
**Question 1**					
*Once, twice or three times a week*	86	61.4 %	53.2 %	69.2 %	3 ± 1
*Never, once a year or some times a year*	54	38.6 %	30.8 %	46.8 %	
**Question 2**					
*Once, twice or three times a week*	105	75.0 %	67.4 %	81.6 %	3 ± 2
*Never, once a year or some times a year*	35	25.0 %	18.4 %	32.6 %	
**Question 3**					
*Absolutely or in general it is true*	138	98.6 %	95.5 %	99.7 %	1 ± 1
*Not sure or in general it is not true*	2	1.4 %	0.3 %	4.5 %	
**Question 4**					
*Absolutely or in general it is true*	131	93.6 %	88.6 %	96.8 %	2 ± 1
*Not sure or in general it is not true*	9	6.4 %	3.2 %	11.4 %	
**Question 5**					
*Absolutely or in general it is true*	125	89.3 %	83.4 %	93.6 %	2 ± 1
*Not sure or in general it is not true*	15	10.7 %	6.4 %	16.6 %	

⁎ Average for a Likert scale of 6 parameters *There may be “missing data”.

**Table 4:** Distribution of the answers on DUREL according to DC location.

	*Once, twice or three times a week*	*Never, once a year or some times a year*	P value
**Q1**			
Colonic	15(17.4 %)	19(35.2 %)	0,090
Ileal	24(27.9 %)	14(25.9 %)	
Ileocolonic	41(47.7 %)	17(31.5 %)	
Perianal	6(7 %)	4(7,4 %)	
**Q2**			
Colonic	28(26.7 %)	6(17.1 %)	0,636
Ileal	27(25.7 %)	11(31.4 %)	
Ileocolonic	42(40 %)	16(45,7 %)	
Perianal	8(7.6 %)	2(5.7 %)	


	*Absolutely or in general it is true*	*Not sure or in general it is not true*	Valor de p
**Q3**			
Colonic	34(24.6 %)	0(0 %)	0,998
Ileal	37(26.8 %)	1(50 %)	
Ileocolonic	57(41.3 %)	1(50 %)	
Perianal	10(7.2 %)	0(0 %)	
**Q4**	138(%)	2(0 %)	
Colonic			0,443
Ileal	34(26 %)	4(44.4 %)	
Ileocolonic	56(42.7 %)	2(22.2 %)	
Perianal	9(6.9 %)	1(11.1 %)	
**Q5**			
Colonic	30(24 %)	4(26.7 %)	0,527
Ileal	32(25.6 %)	6(40 %)	
Ileocolonic	54(43.2 %)	4(26.7 %)	
Perianal	9(7.2 %)	1(6.7 %)	

P value based on qui-quarter or Fischer exact test.

**Fig. 1:**
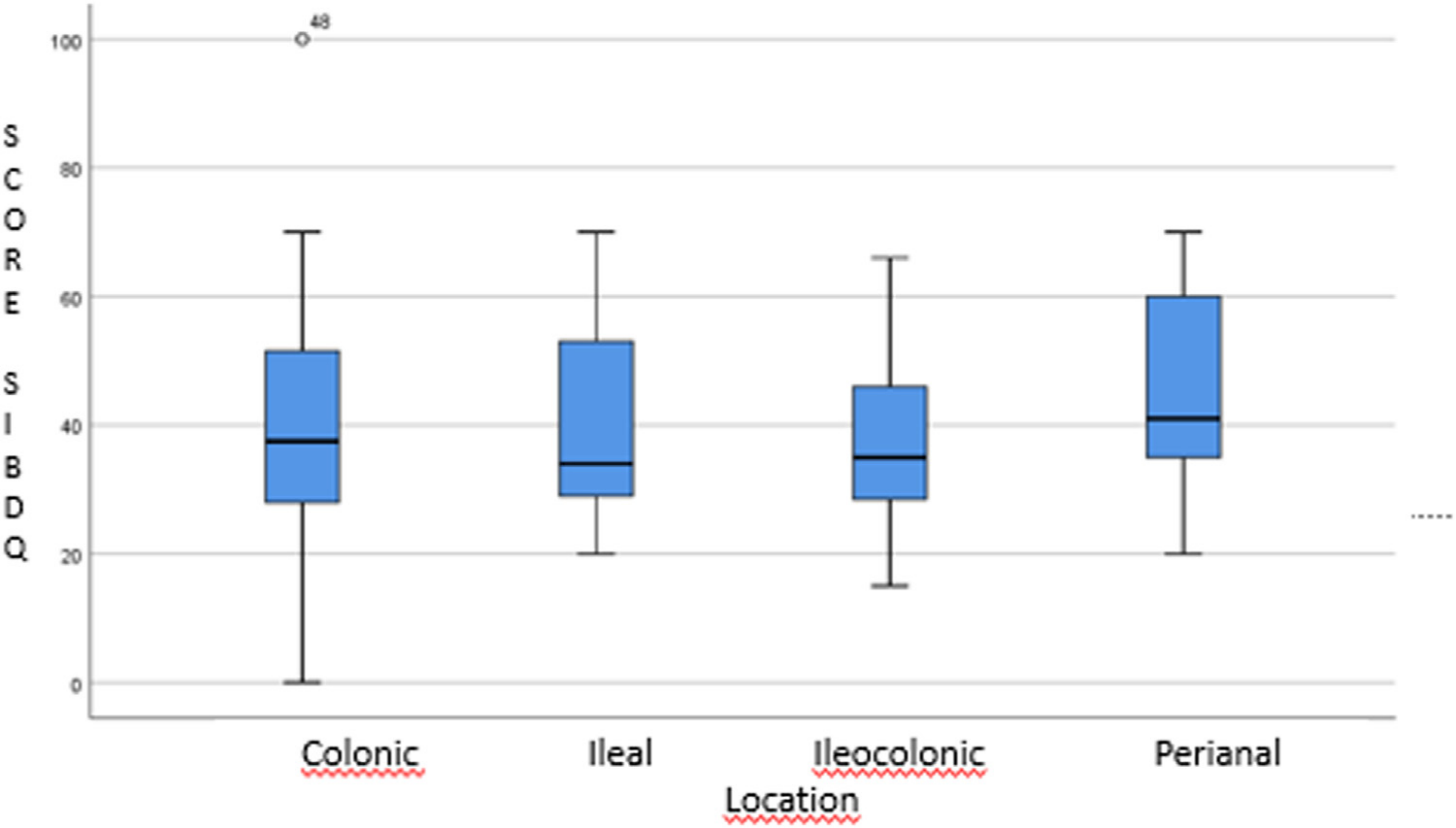
Distribution of the total score SIBDQ according to the location of the CD (p-value based on the KW = 0.786).

The Fig. 1 shows the distribution of the total score SIBDQ according to the location of the CD, which brings the non significant statistical results.

There were also 8 other statistical confrontations that did not reach significance: the first three involving the R/E Index versus each of the CD behaviors (inflammatory, penetrating and fistulizing) and another 5 studies for each of the DUREL questions individually versus the SIBDQ quality of life index. The authors decided to deepen the statistical design in order to confirm the possibilities of significance, which was not observed.

## Discussion

Through the analysis of the results of the socio-demographic questionnaire, the authors observed that the income level (72.6 % up to 3 minimum wages) and the high rate of belonging to a religion (92.4 %) leads us to classify the studied group as homogeneous in these areas. characteristics presented.

The fact that an individual declares himself to belong to a religion does not necessarily refer to the fact that he has a high level of religiosity and spirituality.

In 2017, Panzini brought a new concept of quality of life, which transcends the concept of absence of disease, and covers several domains: physical, psychological, and feeling of mental and spiritual well-being, which, according to individual perception, seeks a cultural context and the purpose of life.^9^

The quality of life assessed by the S-IBDQ questionnaire resulted in a median of 40 out of a total of 70; a minimum of 10 as a very bad QL and a maximum of 70 representing an excellent QoL. In comparison with the scientific literature, the authors observed a great diversity of statistics due to several factors involved, such as the profile of outpatients, service profile as a reference center, clinical phase of patients, psychological reception, adherence to treatment, etc.^18^, ^19^, ^20^

The following statistical correlation studies were performed:

1. Total quality of life score (IBDQ) versus disease location and behavior. There was no statistical significance, showing that regardless of CD location and behavior, patients' quality of life is affected in the same way.
  The initial question was to determine whether the difference in location and/or behavior exposed the patient to a difference in quality of life, a fact that was not materialized in this study.
2. Religiosity/spirituality versus disease phenotypes and behavior (both for the total assessment and for each of the 5 questions of the DUREL index) and correlation between the total score of quality of life versus the answers for R/E (DUREL) and in all results were not observed significance.

The Inflammatory Bowel Disease Outpatient Clinic of the Discipline of Coloproctology at HC FMUSP, due to its complexity and for being an important reference in CD, presents homogeneity in sociodemographic characteristics, quality of life and R/S indexes regardless of phenotypes and behavior, a fact that makes it difficult to statistical analysis, as there are no parameters for comparison.

Since the study was carried out in a follow-up population for Crohn's disease, an important question raised was whether it had a high R/E index at the onset of symptoms or whether the evolution of the disease, with all its uncertainties, insecurities and suffering, led him to change its R/E index.^14^^,^^15^

The results point to the continuity of investigations in the area. They invite further investigation from the cross-study of other factors and other samples. It would be important, for example, to work from a more heterogeneous sample with regard to the religious and sociodemographic profile of the patients. Investigating more deeply the spiritual and religious practices of the groups studied in relation to the treatment experience could also offer another type of understanding of the issue.

## Conclusion

1. The population treated at the Inflammatory Bowel Disease Outpatient Clinic of the Proctology Discipline of HC FMUSP is homogeneous in terms of religious characteristics (religious activity, organizational or not) and intrinsic spirituality present in a high degree.
2. The degree of religiosity/spirituality is independent of CD phenotype and behavior.
3. Positive Religiosity and spirituality do not present a correlation with better quality of life of patients in this study.
4. The quality of life when studied and compared to the disease phenotypes did not present statistically significant results.

## Disclosures

Nothing to disclose.

## Writing assistance

None.

## Grant support

No support assistance for this study was necessary.

## Authors’ contributions

José Luiz Amuratti Gonçalves: Was responsible for the study conceptualization, collected data, manuscript original drafting and editing.

José Jukemura: Drafting, translation and revising of the manuscript critically for important intellectual content.

Carolina Bortolozzo Graciolli Facanali: Contributions to the acquisition, interpretation of data work.

Carlos Frederico Sparapan Marques: Contributions to the acquisition, interpretation of data work.

Rodrigo Ambar Filho: Contributions to the acquisition, interpretation of data work.

Carlos Walter Sobrado: Were involved in conception and design of the study.

Sergio Carlos Nahas: Final editing and reviewing.

All authors contributed to the critical revising and the final approval of the manuscript.

## Conflicts of interest

The authors declare no conflicts of interest.
